# Transcriptome sequencing reveals altered long intergenic non-coding RNAs in lung cancer

**DOI:** 10.1186/s13059-014-0429-8

**Published:** 2014-08-13

**Authors:** Nicole M White, Christopher R Cabanski, Jessica M Silva-Fisher, Ha X Dang, Ramaswamy Govindan, Christopher A Maher

**Affiliations:** Department of Internal Medicine, Division of Oncology, Washington University School of Medicine, St Louis, MO 63110 USA; The Genome Institute, Washington University School of Medicine, St Louis, MO 63110 USA; Alvin J Siteman Cancer Center, Washington University School of Medicine, St Louis, MO 63110 USA; Department of Biomedical Engineering, Washington University School of Medicine, St Louis, MO 63110 USA

## Abstract

**Background:**

Long intergenic non-coding RNAs (lncRNAs) represent an emerging and under-studied class of transcripts that play a significant role in human cancers. Due to the tissue- and cancer-specific expression patterns observed for many lncRNAs it is believed that they could serve as ideal diagnostic biomarkers. However, until each tumor type is examined more closely, many of these lncRNAs will remain elusive.

**Results:**

Here we characterize the lncRNA landscape in lung cancer using publicly available transcriptome sequencing data from a cohort of 567 adenocarcinoma and squamous cell carcinoma tumors. Through this compendium we identify over 3,000 unannotated intergenic transcripts representing novel lncRNAs. Through comparison of both adenocarcinoma and squamous cell carcinomas with matched controls we discover 111 differentially expressed lncRNAs, which we term lung cancer-associated lncRNAs (LCALs). A pan-cancer analysis of 324 additional tumor and adjacent normal pairs enable us to identify a subset of lncRNAs that display enriched expression specific to lung cancer as well as a subset that appear to be broadly deregulated across human cancers. Integration of exome sequencing data reveals that expression levels of many LCALs have significant associations with the mutational status of key oncogenes in lung cancer. Functional validation, using both knockdown and overexpression, shows that the most differentially expressed lncRNA, *LCAL1*, plays a role in cellular proliferation.

**Conclusions:**

Our systematic characterization of publicly available transcriptome data provides the foundation for future efforts to understand the role of LCALs, develop novel biomarkers, and improve knowledge of lung tumor biology.

**Electronic supplementary material:**

The online version of this article (doi:10.1186/s13059-014-0429-8) contains supplementary material, which is available to authorized users.

## Background

Lung cancer is among the leading causes of death worldwide and accounts for greater than 150,000 deaths per year just in the United States, greater than the combination of the next three most common cancers (colon, breast and prostate) [1]. To date, lung cancer research has primarily focused on the deregulation of protein-coding genes to identify oncogenes and tumor suppressors that could serve as diagnostic and therapeutic targets, thereby missing long non-coding RNAs (lncRNAs), which have been shown to play a critical role in tumorigenesis [2,3]. Historical focus on protein-coding genes in disease pathology is due to the relatively recent discovery of lncRNAs, the bias of previous technologies (such as microarrays) towards protein-coding genes, and the lack of sufficient datasets to identify lncRNAs in lung cancer.

As part of the ENCODE project, the GENCODE consortium manually curated 9,277 human lncRNAs [4]. However, current estimates suggest that protein-coding genes may be outnumbered by lncRNAs, many of which have yet to be discovered due to their tissue-specific expression profiles and lower expression levels than coding genes [4]. The tissue-specific nature of lncRNAs suggests they may serve as valuable clinical markers [4–6]. However, until we examine each tumor type more closely, many of these clinically relevant lncRNAs may remain elusive. Transcriptome sequencing, or RNA-Seq, offers an unbiased approach for annotating expressed transcripts [5], as exemplified by the discovery of approximately 1,800 unannotated lncRNAs in a cohort of 102 prostate cancer patients, of which 121 were associated with progression [6].

Although originally regarded as transcriptional noise, several well-described examples indicate that lncRNAs may be essential actors in cancer biology, typically facilitating epigenetic gene repression through chromatin-modifying complexes. Examples include the increased expression of *HOTAIR* in metastatic breast cancer [7], *ANRIL*-induced silencing of p15 in leukemia [8], and *MALAT1* association with metastasis in non-small cell lung cancer [9]. In contrast to these well-described examples, however, only a fraction of lncRNAs have documented roles in tumorigenesis [10–12] and even fewer have been implicated in lung cancer. The most well-characterized lncRNA reported in lung cancer is *MALAT1* (*metastasis-associated lung adenocarcinoma transcript 1*), which is associated with high metastatic potential and poor patient prognosis in non-small cell lung cancer patients with and without metastatic tumors [9,13]. More recent studies have found that the intronic non-coding RNA (ncRNA) lncRNA-LET plays a role in the regulation of hypoxia-mediated metastasis in squamous cell lung carcinoma [14], intronic ncRNA AK126698 confers resistance to cisplatin by targeting the Wnt pathway [15], and the lncRNA *SCAL1* (*smoke and cancer-associated lncRNA-1*) is associated with tobacco-induced lung cancer [16]. These individual studies demonstrate the growing importance of lncRNAs in lung cancer while highlighting the need to systematically identify lncRNAs altered in lung cancer. Given the vast quantity of lncRNAs detected and still being discovered, this represents a unique research opportunity to uncover novel biomarkers and therapeutic targets, and to understand their role in tumor biology.

In our study we harnessed the unbiased view of the transcriptome offered by massively parallel next-generation sequencing platforms to explore the recently emerging class of lncRNAs in lung cancer from 197 lung squamous cell carcinoma and 370 adenocarcinoma tumors. Overall, we were able to detect over 3,000 previously unannotated lncRNAs and identify 111 lncRNAs, termed lung cancer-associated lncRNAs (LCALs), that are strongly differentially expressed between lung tumors and adjacent normal tissue. For orthogonal validation we repurposed publicly available exon array-based data coupled with experimental validation (quantitative real-time PCR (qPCR) and rapid amplification of cDNA ends (RACE)) for a subset of LCALs. To elucidate the tissue specificity of lncRNAs altered in lung cancer we conducted a meta-analysis across an additional 324 tumor and adjacent normal pairs from seven different cancers that were sequenced as part of The Cancer Genome Atlas (TCGA) project. Additionally, we incorporated exome sequencing data from TCGA to identify LCALs that were associated with commonly mutated genes. The most differentially expressed lncRNA, *LCAL1*, was functionally validated and determined to regulate cellular proliferation *in vitro.* In summary, we have systematically characterized lncRNAs that may play a critical role in lung cancer.

## Results

### Identification of novel unannotated transcripts

**Figure 1:**
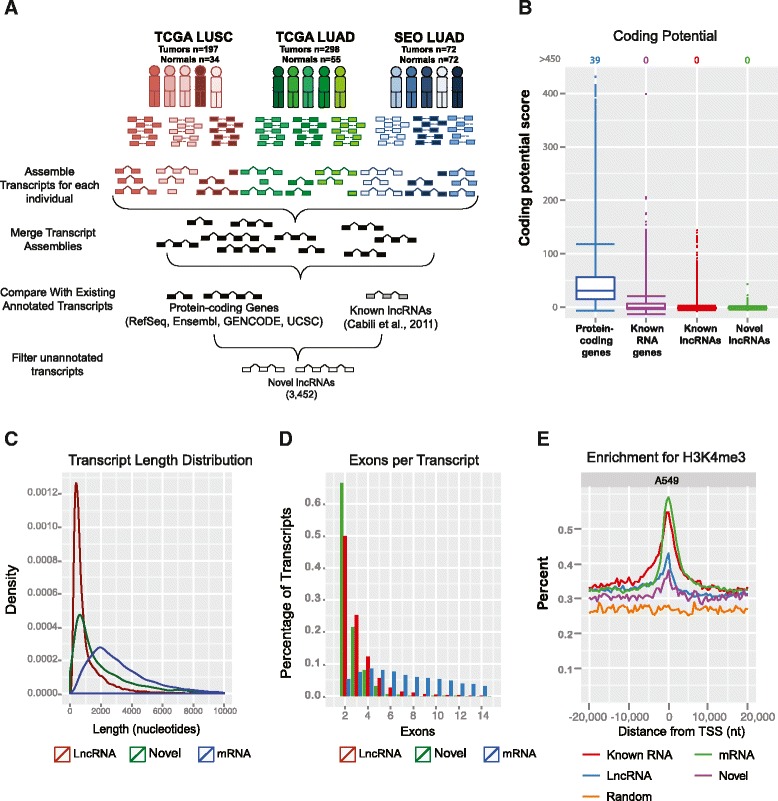
**L** **ncRNA transcript characterization. (A)** Schematic of experimental workflow and RNA-Seq analysis. **(B)** Coding potential of unannotated transcripts using GeneID. Values at the top indicate the number of genes above 450. **(C)** Distribution of transcript lengths for lncRNAs (red), novel transcripts (green), and protein-coding genes (blue). **(D)** Distribution of number of exons per transcript for lncRNAs (red), novel transcripts (green), and protein-coding genes (blue). **(E)** H3K4me3 histone modifications associated with active promoters in A549 cells. nt, nucleotides; TSS, transcriptional start site.

To comprehensively characterize the lncRNA landscape in lung cancer we analyzed poly-A purified RNA-Seq data from three cohorts: (1) 197 squamous cell carcinomas with 34 matched adjacent normal from TCGA [17] (LUSC cohort); (2) 298 adenocarcinomas with 55 matched adjacent normal from TCGA (LUAD cohort); and (3) 72 adenocarcinomas and adjacent normal pairs from a Korean population [18] (Seo cohort). To identify novel unannotated transcripts, the aligned reads for each sample underwent *de novo* assembly using Cufflinks [19] and were subsequently merged together into a consensus lung cancer transcriptome (Figure 1A). As none of these data sets utilized stranded library protocols, we were prevented from discriminating any regions in which two independent transcripts overlap. Therefore, we focused solely on intergenic transcripts (as described in Materials and methods). To ensure that transcripts were not previously annotated, the consensus lung transcriptome was compared against a comprehensive gene database comprised of UCSC [20], Ensembl [21], GENCODE [22], and RefSeq [23] as well as a set of lncRNAs in human development [5]. To remove extensions of annotated transcripts, we filtered any transcript intersecting a protein-coding exon. Last, transcripts lacking a splice junction, and therefore could be due to potential DNA contamination, or less than 200 nucleotides in length were filtered. This resulted in the discovery of 3,452 multi-exon genes residing within intergenic regions of the genome (Table S1 in Additional file 1).

### Characterization of novel lncRNAs

To ensure that the novel candidates that we predicted did not encode proteins, we used GeneID [24] and CPAT [25] to measure (1) the protein-coding potential and (2) the ORF size in each lncRNA sequence. For comparison, genes were classified into four categories: (i) unannotated transcripts (Novel); (ii) non-coding RNAs annotated by RefSeq (Known_RNA); (iii) protein-coding genes annotated by RefSeq (mRNA); and (iv) previously annotated lncRNAs (lncRNAs) [5]. The unannotated transcripts have a lower coding potential and ORF length relative to protein-coding genes but similar coding potential to known RNA genes and recently reported lncRNAs (Figure 1B; Figure S1A,B in Additional file 2; Table S2 in Additional file 1)*.* Additionally, the expression levels of the novel unannotated transcripts were skewed towards lower expression, which was also observed with annotated RNAs and recently discovered lncRNAs (Figure S1C in Additional file 2). In addition to expression levels, the transcript characteristics of the novel lncRNAs mimic previously reported lncRNAs. As shown in Figure 1C, the overall transcript length of the novel lncRNAs (median 1,823 nucleotides) is shorter than protein-coding genes (2,757 nucleotides; *t*-test, *P*-value <5.4 × 10^-8^), which is expected given the bias of lncRNAs having fewer exons than protein-coding genes (Figure 1D).

It was recently found that transposable elements significantly contributed to the origin, diversification, and regulation of lncRNAs in human and vertebrates [26,27]. Consistent with earlier reports [26,27], we also found that repetitive elements accounted for 30.2% of the novel lncRNAs, with the most abundant families including LINE/L1, LINE/L2, SINE/Alu, SINE/MIR, and LTR/ERVL-MaLR (Figure S1D in Additional file 2).

To determine whether the predicted novel lncRNAs are independent transcripts rather than extensions of neighboring protein-coding transcripts [28], we leveraged existing ENCODE ChIP-Seq data available for the H3K4me3 histone modification that is associated with active promoters. We focused on the epithelial cell line (A549) derived from a lung carcinoma tissue to better reflect our tumor tissue cohort. We observed enrichment for histone modifications characterizing transcriptional start sites and active transcription (Figure 1E). Protein-coding transcripts had the highest enrichment, with recently discovered lncRNAs [5] and novel lncRNAs showing nearly equivalent profiles. Taken together, characterization of the unannotated transcripts suggests that they are novel lncRNAs.

### Altered lncRNA expression in lung cancer tissues relative to adjacent normal lung tissues

**Figure 2:**
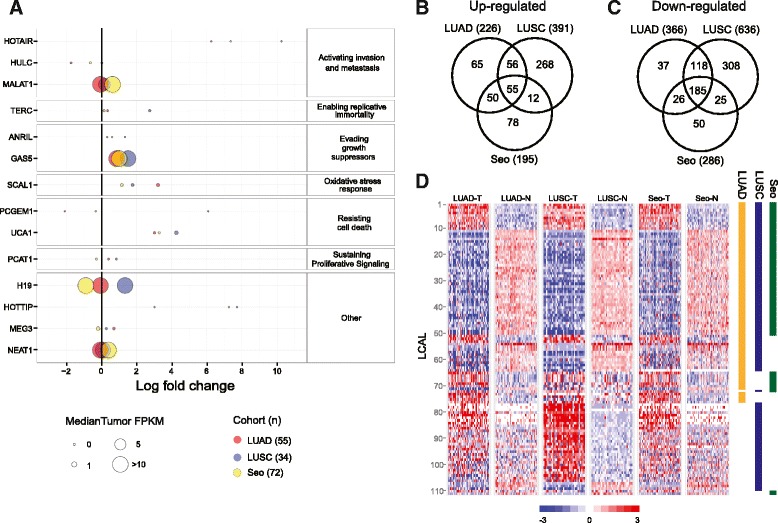
**Altered lncRNAs across lung cancer subtypes. (A)** Expression levels of lncRNAs with known oncogenic function across three lung cohorts, denoted by different colors. The size of each point is proportional to average FPKM expression across the tumors for up-regulated lncRNAs or across the normal tissues for down-regulated lncRNAs. The x-axis shows log_2_ fold change of tumor relative to normal. **(B,C)** Venn diagrams showing the overlap of significantly up-regulated lncRNAs (B) and down-regulated lncRNAs (C). **(D)** Expression levels of tumor and normal samples for each cohort across the 111 LCALs. Colored bars to the right designate in which cohort(s) a given LCAL is differentially expressed.

An initial investigation of well-characterized lncRNAs across cancers (reviewed in [2,3]) revealed that most lncRNAs with known oncogenic function either do not appear to be altered in our lung cohorts or are very lowly expressed (Figure 2A). For instance, although *HOTAIR* appears to have a strong log fold change between tumor and normal tissues, its median tumor expression level is <0.1 FPKM (fragments per kilobase of transcript per million mapped reads). Therefore, we sought to identify lncRNAs showing significant expression differences between tumors and normal lung tissues in each of the three cohorts. Before testing for differential expression, we applied a series of filtering steps (see Materials and methods) to focus on intergenic non-coding RNAs displaying reliable expression levels across a majority of the samples (Figure S2A in Additional file 2). We identified 1,027 differentially expressed lncRNAs in LUSC, 592 in LUAD, and 481 in Seo (Tables S3 to S5 in Additional file 1; Figure S2B-D in Additional file 2). Of these, 240 were commonly differentially expressed in all three cohorts (55 up- and 185 down-regulated; Figure 2B,C).

Using the results from all three cohorts, we composed a list of 111 intergenic lung cancer-associated lncRNAs (LCALs) that represent the most highly expressed and differentially expressed transcripts (Figure 2D; Table S6 in Additional file 1). Fifty LCALs were differentially expressed in all three cohorts, 22 in two cohorts, and 39 unique to a single cohort. Not surprisingly LUSC had the most cohort-specific lncRNAs, as it is the only squamous cell lung cancer cohort in the study. Additionally, 57 LCALs were differentially expressed in both adenocarcinoma cohorts and 21 were differentially expressed in a single adenocarcinoma cohort. The differences between the LUAD and Seo lncRNAs may represent differences in the ethnic backgrounds amongst the patient population since the Seo cohort is an exclusively Korean patient population.

The 111 LCALs include a lncRNA known to play a role in lung cancer (*SCAL1* [16]), cancer-associated lncRNAs not previously implicated in lung cancer (*CCAT1* [29], *ESCCAL-1* [30], *LINC00261* [31], *linc-UBC1* [32], *UCA1* [33], ENST00000547963 [34], and *PART-1* [35]), a lncRNA implicated in a lethal lung developmental disorder (*FENDRR* [36]), and three previously unannotated lncRNAs. Interestingly, the remaining 99 lncRNAs were previously annotated in normal human tissues but not implicated in human disease.

### LncRNAs associated with lung cancer subtypes

Lung cancer is a heterogeneous disease comprised of different subtypes and molecular aberrations. Therefore, we next sought to better understand the role of lncRNAs in each subtype. We found 463 and 315 up- and down-regulated genes, respectively, in LUAD tumors relative to LUSC (Table S7 in Additional file 1; Figure S3 in Additional file 2). Of the 50 LCALs that differentiated tumor from normal tissues across all three cohorts, 27 were differentially expressed between LUAD and LUSC tumors. This subset of LCALs could potentially serve as important biomarkers for lung cancer due to their differential expression between tumor and normal lung tissue as well as between adenocarcinoma and squamous cell carcinoma tumors.

### Orthogonal validation of altered lung adenocarcinoma lncRNAs using Affymetrix exon arrays

To provide additional independent validation of altered lncRNA expression, we repurposed the existing Affymetrix Human Exon 1.0 ST array with publicly available expression profiling data from an independent cohort of 20 adenocarcinoma lung cancer patient tumor and adjacent normal samples collected at the University of Pittsburgh (Gene Expression Omnibus accession GSE12236) [37]. In total, 81.25% of all lncRNAs were covered by at least one probeset overlapping an exon (including 57.9% of the 3,246 novel lncRNAs). Of the 111 LCALs, 98 (88.3%) were covered by at least one probeset. This demonstrates that although the Human Exon Array is able to measure expression levels of a large number of lncRNAs, it does not provide the same genome-wide coverage as RNA-Seq and therefore misses potentially informative lncRNAs.

**Figure 3:**
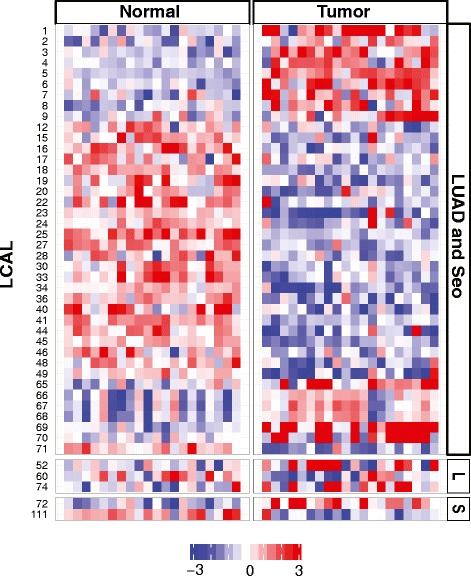
**Independent validation of lung cancer-associated lncRNAs.** Heatmap of 45 LCALs confirmed as differentially expressed in lung adenocarcinoma tumor and matched normal tissues by Human Exon Array. LCALs are grouped by the RNA-Seq cohort in which they were called significant: LUAD and Seo, LUAD only (denoted 'L'), and Seo only (denoted 'S').

Next, we wanted to determine whether the LCALs were also differentially expressed in the adenocarcinoma array data. We restricted our analysis to 66 LCALs that were covered by at least one probeset and differentially expressed in at least one of the adenocarcinoma cohorts. The array confirmed differential expression of 45 of the 66 (68.2%) LCALs (Figure 3; Table S8 in Additional file 1). This validation rate increased to 83.3% (40/48) when considering LCALs called differentially expressed in both adenocarcinoma cohorts.

### Experimental validation of LCALs in cell lines and an independent tissue panel

**Figure 4:**
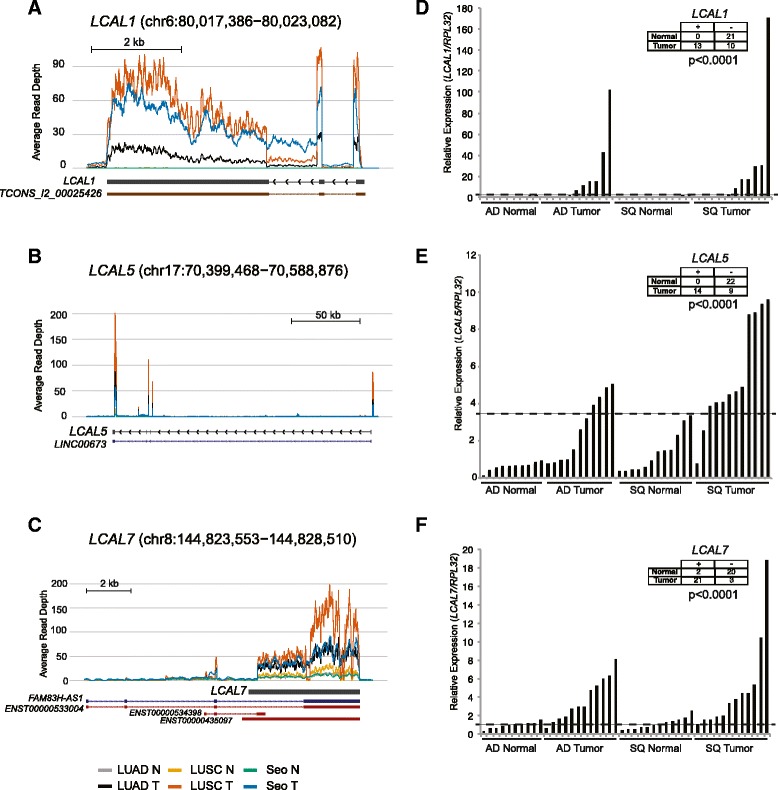
**LCAL expression in lung cancer. (A-C)** Coverage maps showing the average expression levels of tumor and normal samples across all three lung cancer cohorts for *LCAL1* (A), *LCAL5* (B), and *LCAL7* (C)*.* Annotated RefSeq (dark blue), Ensembl (red), Human Body Map lncRNAs (brown), and full-length transcripts as determined by 5’ and 3’ RACE in H322M cell line (black) are shown below each plot. **(D-F)** qPCR validation in an independent cohort of human adenocarcinoma and matched controls and squamous cell carcinoma and matched controls for *LCAL1* (D), *LCAL5* (E), and *LCAL7* (F)*.* Insert tables distinguish ‘high’ and ‘low’ expression of *LCALs* in tumors using the value as denoted by the dotted line.

To further confirm alterations of lncRNA expression in lung cancer, we validated a subset of LCALs across a panel of lung cancer cell lines by qPCR (Figure S4 in Additional file 2). Moreover, we confirmed the cancer-specific expression of the six lncRNAs by qPCR in an independent cohort of lung tissues, collected at Washington University, comprised of adenocarcinoma with matched control tissue and squamous cell carcinoma and matched control tissue (Figure 4; Figure S5 in Additional file 2). This independent cohort confirmed the subtype-specific expression of *LCAL80* and *LCAL85* (Figure S5E,F in Additional file 2).

LncRNAs are known to display features typical of transcription by RNA polymerase II, including 5′ capping, 3′ polyadenylation, and intron splicing [38]. However, despite observing H3K4me3 marks, indicative of promoter regions for the novel lncRNAs, we were still concerned that the lower expression levels of lncRNAs would poorly define the transcript boundaries. Therefore, to characterize the lncRNA transcripts and ensure that we observe the full-length transcript, we designed gene-specific primers for four lncRNA genes and conducted 5′ RACE and 3′ RACE using Invitrogen’s Gene Racer Kit. In each instance we were able to recapitulate a full-length transcript corresponding to the observed RNA-Seq coverage (Figure 4; Figure S5 in Additional file 2).

### Aberrantly expressed lncRNAs across human cancers

**Figure 5:**
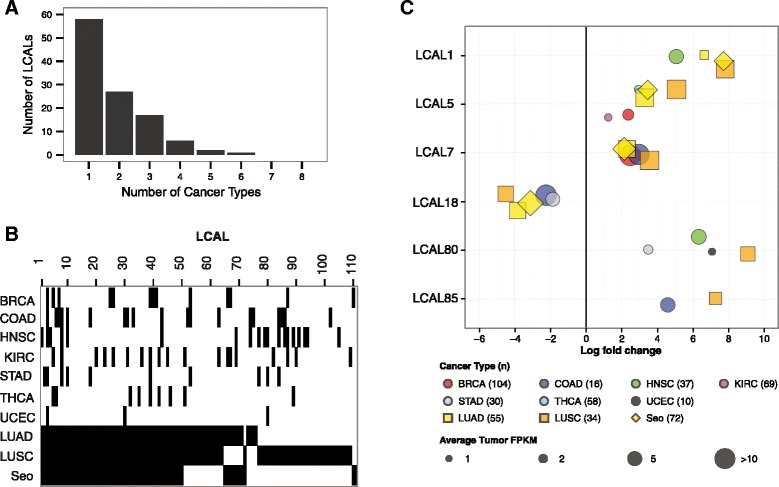
**Aberrantly expressed lncRNAs across human cancers. (A)** Distribution of the number of cancer types in which LCALs are differentially expressed. All lung cohorts were considered as one cancer type, and x = 1 corresponds to differentially expressed in lung only. **(B)** Heatmap showing the distribution of differentially expressed LCALs across the three lung cohorts and seven additional TCGA cohorts. Black bars designate that an LCAL is differentially expressed in a given cancer. **(C)** Expression levels and fold changes for six LCALs that were experimentally validated. The size of each point is proportional to average FPKM expression across the tumors for up-regulated lncRNAs or across the normal tissues for down-regulated lncRNAs. Colors and symbols correspond to cancer type. Only cancer types in which the LCAL is differentially expressed are plotted. BRCA, breast invasive carcinoma; COAD, colon adenocarcinoma; HNSC, head and neck squamous cell carcinoma; KIRC, kidney renal clear cell carcinoma; STAD, stomach adenocarcinoma; THCA, thyroid carcinoma; UCEC, uterine corpus endometrial carcinoma.

We next investigated whether the identified LCALs have tissue-specific expression profiles, ideal for a putative biomarker, or are altered across numerous human cancers, suggesting that they may have a more common oncogenic or tumor suppressive role in multiple cancers. We conducted a pan-cancer analysis of RNA-Seq data from 324 matched tumor and adjacent normal pairs from seven additional TCGA solid tumor types (breast invasive carcinoma [39], colon adenocarcinoma [40], head and neck squamous cell carcinoma, kidney renal clear cell carcinoma [41], stomach adenocarcinoma, thyroid carcinoma, and uterine corpus endometrial carcinoma [42]). We found that 52.3% (58/111) of LCALs were specific to lung cancer and 24.3% (27/111) were differentially expressed in only one additional cancer type (Figure 5A,B). This demonstrates that most LCALs are specific to lung cancer and thus may have potential use as tissue-specific biomarkers.

We further investigated LCALs that were altered across multiple cancers. Of the nine LCALs that were altered in at least three additional cancers, only *LCAL84* has been previously studied in cancer. *LCAL84* (ENST00000547963) is a member of a three-lncRNA signature associated with the survival of patients with esophageal squamous cell cancer [34]; thus, it is not unexpected that it is differentially expressed in the two squamous cell cohorts, head and neck and lung, although it is also differentially expressed in colon and stomach adenocarcinoma. Two of the experimentally validated LCALs, *LCAL5* and *LCAL80*, were also broadly altered across three additional cancers (Figure 5C). This meta-analysis emphasizes the potential significance of previously uncharacterized lncRNAs across multiple cancers.

### Associations with mutation status

**Figure 6:**
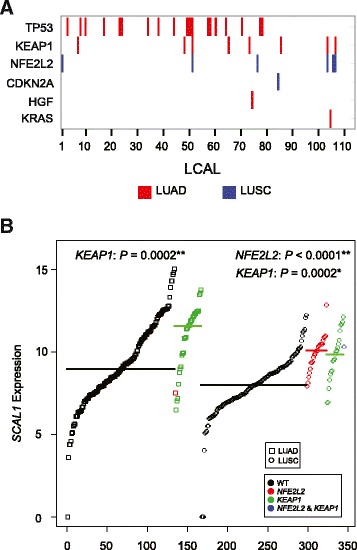
**Association between LCAL expression and mutation status. (A)** Significant associations between LCAL expression and mutation status. Red bars designate significant associations (false discovery rate (FDR) <0.01) across LUAD tumors and blue bars across LUSC tumors. **(B)** Expression levels of *SCAL1* (*LCAL51*), measured by log_2_ FPKM, for wild-type (WT; black), *NFE2L2* mutant (red), *KEAP1* mutant (green), and both *NFE2L2* and *KEAP1* mutant (blue) samples. Data points are ordered by expression levels and symbols designate cohort (squares for LUAD, circles for LUSC). Thick colored lines represent the median expression levels across each group. *P*-values for each mutational association are also reported (*FDR <0.05, **FDR <0.01).

A recent study demonstrated the impact of oncogene-activating mutations on lncRNAs [43]. Therefore, to determine if LCAL expression levels are associated with mutational status we focused on 16 protein coding genes that have been reported by TCGA as mutated in at least 10% of lung cancer tumors [44]. We tested each TCGA lung cohort separately due to differences in the mutational frequencies between the subtypes. In LUAD, *TP53* and *KEAP1* mutational status are associated with 19 and 8 LCALs, respectively. In LUSC, *NFE2L2* mutational status is associated with six LCALs (Figure 6A). None of the remaining mutations in either LUAD or LUSC had an association with more than a single LCAL. The mutational status of *NFE2L2* and *KEAP1*, which have been shown to regulate cell response to oxidative damage [45], is associated with expression levels of multiple LCALs, including *LCAL51*, or *SCAL1* (Figure 6B). Additional significant associations with *TP53*, *NFE2L2*/*KEAP1*, *CDKN2A*, and *HGF* are shown in Figure S6 (Figure S6 in Additional file 2).

### Characterization of *LCAL1*

To determine if the lncRNAs found in this study have phenotypic consequences, we chose to examine the most differentially expressed lncRNA, *LCAL1*, in both adenocarcinoma and squamous cell carcinoma. *LCAL1* is located on chromosome 6q14.1 and produces a three-exon transcript (Figure 4A). ENCODE data show DNaseI hypersensitivity and transcription factor binding upstream of *LCAL1*, suggesting regulatory activity within the *LCAL1* promoter (Figure S7 in Additional file 2). Interestingly, *LCAL1* lacks strong base pair conservation, using PhyloP. However, *LCAL1* appears to be evolutionarily conserved amongst primates, suggesting a more recent evolution (Figure S7 in Additional file 2). Subcellular localization revealed that *LCAL1* was enriched in the nucleus, which is common amongst lncRNAs associated with gene regulation [4] (Figure S8 in Additional file 2).

**Figure 7:**
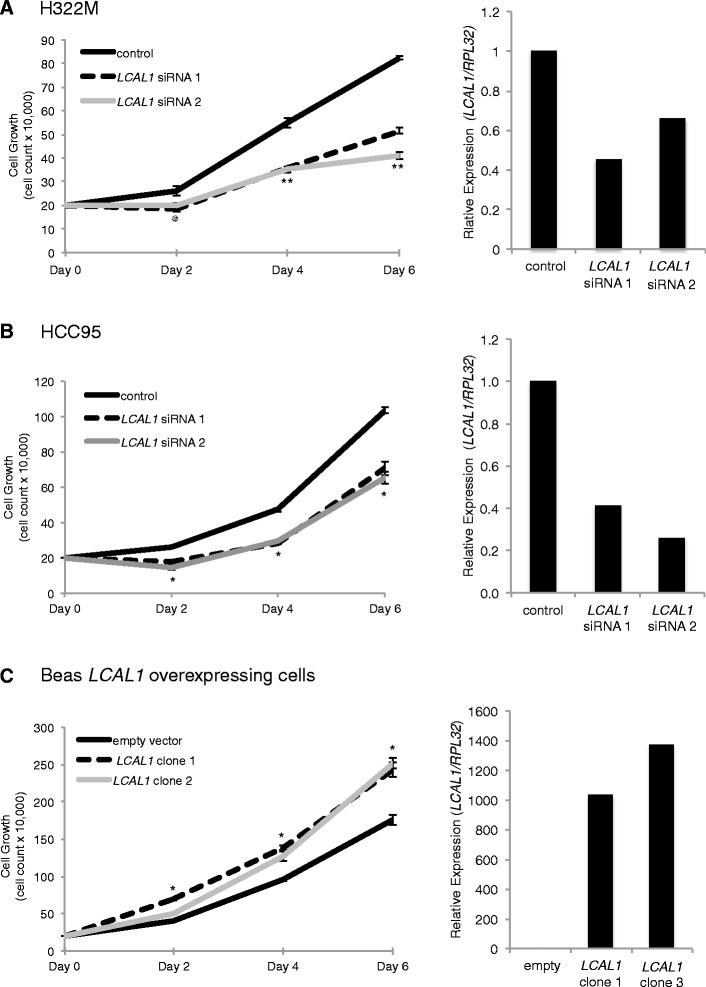
***LCAL1*** **expression affects cell growth. (A, B)** Cell proliferation assay in H322M (A) and HCC95 (B) cells using *LCAL1* siRNAs. qPCR validation of *LCAL1* siRNA knockdown is shown on the right. **(C)** Cell proliferation assay in BEAS-2B overexpressing clones of *LCAL1* with qPCR validation of *LCAL1* expression in BEAS-2B cells on the right. ^@^ *P* ≤ 0.05, *P ≤ 0.01, **P ≤ 0.001 by a two-tailed Student’s *t*-test. The same significance applies for siRNA 1 and siRNA 2 at all time points. All error bars are mean ± standard error of the mean across *n* = 3 biological replicates in two independent experiments.

Next, we wanted to assess the functional significance of *LCAL1*. Short interfering RNAs (siRNAs) were designed to help assess the function of *LCAL1* in lung cancer. Greater than 50% knockdown of *LCAL1* in the cell line H322M, which models adenocarcinoma, with two different siRNAs resulted in decreased cell growth as measured by cell counting for six days. Both *LCAL1* siRNA knockdowns in H322M caused at least a 24% decrease in cell growth starting at day 2 and a 37% or 50% decrease in cell growth in siRNA 1 or siRNA 2, respectively, at day 6 compared to control cells (Figure 7A). In our original panel of nine different cancer cell lines, *LCAL1* was only highly differentially expressed in one cell line; therefore, we screened additional squamous cell carcinoma lines and found *LCAL1* to be highly differentially expressed in HCC95 (Figure S9 in Additional file 2). Greater than 50% knockdown of *LCAL1* in HCC95 cells recapitulated cell growth observations in the H322M cell. Both siRNA knockdowns in HCC95 caused at least a 30% decrease in cell growth starting at day 2, which was maintained through to the end of the experiment at day 6 compared with control cells (Figure 7B). Furthermore, stable overexpression of *LCAL1*, using two different clones, in the control cell line BEAS-2B showed a significant increase in cellular proliferation starting on day 2 and continuing until the end of the experiment at day 6 with a 38% and 43% growth increase, respectively (Figure 7C). Overexpression of *LCAL1* in normal BEAS-2B cells, at physiological levels in human tumors, is proof of principle that this lncRNA is sufficient to affect cellular growth independently of other common cancer mutations, thus highlighting the importance of *LCAL1* in lung cancer biology.

To confirm that changes in cell growth were due to a proliferative effect of *LCAL1* expression, Alamar Blue proliferation experiments were also conducted. After 72 hours *LCAL1* knockdown cells were replated and Alamar Blue reduction was assessed on days 2, 4, and 6. We see a similar significant decrease of proliferation in both siRNA constructs compared with scrambled control in both H322M and HCC95 cell lines (Figure S10 in Additional file 2). In addition, there was no change in apoptosis or necrosis in both cell lines with decreased *LCAL1* expression compared with control as measured by annexin V and propidium iodide staining at 72 hours post-knockdown (data not shown). These results highlight the biological importance of *LCAL1* in promoting tumorigenesis.

## Discussion

The utilization of lncRNAs as biomarkers, and more recently active tumorigenic factors influencing protein function, demonstrates the necessity for more extensive studies characterizing and understanding the role of lncRNAs in disease progression. In this study we used an unbiased approach to systematically categorize lncRNAs in 567 tumors from three separate publicly available RNA-Seq lung data sets. We identified 111 intergenic lung cancer-associated lncRNAs, or LCALs, most of which were not previously implicated in cancer development and progression. Further stratification of the 111 LCALs determined 27 LCALs to be subtype-specific, and therefore might serve as important biomarkers to form a molecular signature in stratifying adenocarcinoma and squamous cell carcinoma. A meta-analysis across seven additional cancers established that most (over 50%) LCALs appear to have restricted expression in lung cancer, suggesting they may be involved in disease pathogenesis and serve as putative biomarkers. Moreover, a small percentage of LCALs are highly differentially expressed in at least one other cancer, with nine being expressed in at least three additional cancers. This analysis highlights the importance of lncRNAs not only in lung cancer but also as broad oncogenic factors and lays the groundwork for future studies to determine the mechanisms by which these newly discovered non-coding RNAs act in cancer progression.

In our study we provided a comprehensive analysis to detect novel lncRNAs across lung cancer patients that led to the annotation of over 3,000 novel lncRNAs. However, to ensure that we were annotating high-confidence candidates we focused on multi-exon genes. Additionally, the publicly available data collections used for this study did not utilize stranded libraries and therefore did not allow for accurate annotation of antisense non-coding RNAs. Furthermore, the data used in this study focused on polyA+ RNA and therefore may have missed some non-coding RNAs. However, for the first time we were able to identify solid tumor-associated lncRNAs not previously implicated in lung cancer as well as uncharacterized lncRNAs altered in lung cancer. For example, *linc-UBC1* (*LCAL6*) was discovered in bladder cancer [32]; *UCA1* (*LCAL52*) in bladder [33], ovarian [46] and breast cancer [47]; *LINC00261* (*LCAL62*) in gastric cancer [31]; *ESCCAL-1* (*LCAL80*) [30] and *ENST00000547963* (*LCAL84*) [34] in esophageal squamous cell carcinoma; *CCAT1* (*LCAL85*) in colon cancer [29]; and *PART1* (*LCAL92*) in prostate cancer [35] and glioblastoma multiforme [48]. Overall, these findings emphasize the importance of unbiased sequencing approaches to better understand the non-coding RNA landscape of cancer.

One of the major challenges for studying lncRNAs is to determine their potential functional role. Interestingly, mutations in well-established oncogenes have shown association with lncRNAs. For example, the lncRNA *BANCR* was found as a recurrently overexpressed transcript in *BRAF*^V600E^-mutant human melanoma, which is the most activating mutation in melanoma, with a potential role in regulating cell migration [43]. Additional studies have also established that lncRNAs, such as *lncRNA-p21*, contain functional p53-binding motifs [49], indicating these lncRNAs serve as transcriptional targets in key biological pathways. Here we discovered that some LCALs are also associated with mutational status, thereby implicating them in key oncogenic pathways. In addition to altered LCAL expression associating with *TP53* mutation status, some of the LCALs also associated with mutations in *KEAP1* and *NFE2L2*, which are key players in the oxidative stress pathway. For instance, *SCAL1* (*LCAL51*) was found to be associated with *KEAP1* mutation status in LUAD and *NFE2L2* mutation status in LUSC (Figure 6B) and was recently shown to act downstream of NRF2 and mediate oxidative stress protection in airway epithelial cells [16]. The association of *SCAL1* with oxidative stress, which has been previously explored through experimental validation [16], further supports that the association of LCAL expression with mutational status can potentially elucidate their function and serve as the basis for future cancer biology studies.

To determine the importance of these LCALs in lung disease pathology, we proceeded with functional studies of *LCAL1*, the top up-regulated lncRNA in both LUAD and LUSC. Cellular proliferation studies revealed an oncogenic phenotype, as shown by siRNA knockdown studies of *LCAL1* resulting in decreased cellular growth in two cellular models of lung cancer, a non-small cell lung carcinoma cell line (H322M) and a squamous cell carcinoma cell line (HCC95). Moreover, as proof-of-principle, our *LCAL1* overexpression studies highlight increased proliferation compared with control BEAS-2B empty vector cells, suggesting that altered *LCAL1* is sufficient for promoting the etiology of the disease. Furthermore, our *LCAL1* experiments highlight the potential functional contribution additional LCALs may have in various facets of lung tumorigenesis.

## Conclusions

To date, lung cancer research has primarily focused on the deregulation of protein-coding and microRNA genes to identify oncogenes and tumor suppressors as potential diagnostic and therapeutic targets. However, lncRNAs represent an emerging and under-studied class of transcripts that have a significant role in human cancers. This study leverages RNA-Seq data from approximately 550 patient specimens representing an unmatched lung cancer transcriptome analysis to date to discover 111 lung cancer-associated lncRNAs (LCALs). We have experimentally validated a subset of LCALs and demonstrated that the most commonly up-regulated lncRNA across lung subtypes, *LCAL1*, contributes to cellular proliferation. A meta-analysis across human cancers revealed a subset of LCALs that have restricted expression and may represent putative biomarkers while a subset appear to be altered in multiple solid tumors, suggesting a common oncogenic role. Taken together, our study highlights the comprehensive scope of lncRNAs (both previously known and novel) that may contribute to lung cancer. While we already demonstrate the biological significance of *LCAL1*, our study provides a framework for subsequent research exploring additional LCALs in lung tumorigenesis as well as assessing their prognostic and predictive potential.

## Materials and methods

### Lung RNA-Seq datasets

Raw sequences from three previously sequenced lung RNA-Seq datasets were downloaded: (1) 72 adenocarcinoma tumor and adjacent normal pairs [18] (referred to as 'Seo') from EBI-SRA under accession number ERP001058; (2) 55 adenocarcinaoma tumors and adjacent normal pairs, plus an additional 243 unmatched tumors, from TCGA (referred to as 'LUAD'); and (3) 34 squamous cell carcinoma tumors and adjacent normal pairs, plus an additional 163 unmatched tumors, from TCGA [17] (referred to as 'LUSC'). Sequence reads were aligned using TopHat v1.3.0 [50].

### Discovery of unannotated lncRNAs

All available samples (adjacent normal, matched tumor, unmatched tumor) from the LUAD, LUSC and Seo cohorts were used to discover novel expressed transcripts. Transcript assemblies were generated using Cufflinks v2.0.2 [19] in *de novo* mode and subsequently merged together with Cuffmerge to generate a consensus transcriptome across the cohort. To identify unannotated transcripts, a comprehensive set of protein-coding gene annotations was generated by downloading RefSeq, UCSC, Ensembl and GENCODE v17 gene annotations, in gene transfer format (GTF) and aggregated together (each downloaded on 20 September 2013). Additionally, the lncRNAs identified from the Human Body Map project were downloaded from UCSC and aggregated to the protein-coding GTF. Cuffcompare was used to compare the lung cancer consensus transcriptome with our comprehensive protein-coding and lncRNA gene reference. The Cuffcompare results were filtered for gene loci that were classified as unannotated (‘u’) and none of the transcripts overlapped an existing gene annotation. This subset was defined as ‘novel transcripts’. Analysis of coding potential of lncRNAs was performed on transcript sequences using GeneID [24] and CPAT and were both pre-trained for human genes [25].

Enrichment for H3K4me3 histone modifications in lung cancer cells was conducted using ENCODE ChIP-Seq data downloaded from the UCSC browser tracks. Coverage was aggregated across 500 nucleotide bins for 20 kb upstream and downstream of the transcription start site for each transcript in the following categories: (i) protein-coding, (ii) known RNAs (reported in RefSeq), (iii) lncRNAs recently annotated in human development, (iv) novel lncRNAs found in this study, and (v) random. The random transcriptional start sites were determined by selecting genomic coordinates randomly throughout the genome.

### Repetitive element analysis of lncRNAs

RepeatMasker annotation for human genome (hg19 assembly) was downloaded from the UCSC database [20]. BEDTools intersect [51] was used to identify overlap between repetitive elements and transcript exons. Repetitive elements that overlapped at least 10 nucleotides with an exon were considered for further analysis. Anything that was not classified as a transposable element (such as low complexity, satellites, and simple repeats) was removed from further analysis.

### Gene expression analysis

Figure S2A in Additional file 2 shows the multiple steps of our differential expression pipeline. A custom annotation file comprised of lncRNAs from multiple sources was generated by merging noncoding transcripts from GENCODE v17, Ensembl, UCSC, Human Body Map, and our novel lncRNAs. All single-exon transcripts were removed. This list was then merged with all RefSeq noncoding transcripts, including single-exon transcripts, and all transcripts less than 200 nucleotides were removed. Transcripts overlapping an exon from a RefSeq protein coding gene or Ensembl pseudogene were removed, resulting in 34,308 unique transcripts spanning 14,091 gene loci. Gene expression FPKM values were calculated with Cufflinks v2.0.2 using this custom lncRNA annotation file. Additionally, a table comprising read counts for each transcript was calculated using BEDTools version 2.17.0 [51]. We removed lowly expressed transcripts (at least 75% of samples had FPKM <0.1 or read count <25). The set of remaining transcripts was reduced to a set of non-overlapping regions (or 'genes') by comparing all overlapping transcripts and keeping the transcript with the largest average FPKM across all samples as the representative transcript for that region. After TMM normalization [52], edgeR version 3.0.8 [53] was used to identify differentially expressed transcripts between tumor and normal pairs using a matched pair design for the Seo, LUAD and LUSC datasets using cutoffs of false discovery rate (FDR) ≤10^-5^ and absolute fold change ≥2. To obtain the list of LCALs, we selected lncRNAs for which the following criteria all held across at least one cohort: (1) differentially expressed, (2) highly expressed (average tumor or normal FPKM ≥2), and (3) large fold change between tumor and normal (fold change ≥8).

The same pipeline was used for discovering differentially expressed lncRNAs between tumor subtypes. The only difference was that instead of using a matched pair design, we tested for a difference between subgroups after adjusting for gender (LUAD, *n* = 297; LUSC, *n* = 196; two samples without gender information were removed). Heatmaps were generated for each dataset using standardized values by subtracting the median and dividing by the median absolute deviation of each lncRNA. Rows (lncRNAs) were clustered using Ward’s method.

### Expression levels of validated LCALs

For the six LCALs that were experimentally validated (LCALs 1, 5, 7, 18, 80, and 85), coverage across the transcript was calculated by counting the read depth at each base using custom perl scripts and the Bio::DB::Sam BioPerl package. Coverage maps shown in Figure 4 and in Figure S5 in Additional file 2 were created in R using SigFuge version 1.1.2 [54]. For the tables shown in Figure 4 and in Figure S5 in Additional file 2, the cutoff for classifying samples as high or low expression was determined by maximizing the Matthews correlation coefficient [55] and two-sided *P*-values were calculated using Fisher’s exact test.

### Human exon array validation

Affymetrix Human Exon 1.0 ST Array data for 20 lung adenocarcinoma tumor and adjacent normal pairs [37] were downloaded from Gene Expression Omnibus (GSE12236). We chose to repurpose this array platform because it has the most comprehensive probe coverage of lncRNA genes. The genomic coordinates for each probeset were converted from hg18 to hg19 using the UCSC Genome Browser LiftOver tool [56]. Probesets overlapping exons of lncRNAs were determined using custom perl scripts. For annotated lncRNAs, only probesets on the correct strand were used. For the novel lncRNAs where the strand is unknown, overlapping probes on either strand were used. The raw CEL files were processed using Affymetrix Power Tools [57] with RMA normalization [58] to generate transcript-level intensity estimates. For each LCAL that was called differentially expressed in either adenocarcinoma cohort (LUAD or Seo), a paired Wilcoxon signed rank test was performed. LCALs with *P* < 0.05 were considered to be validated by the array.

### Aberrantly expressed lncRNAs across human cancers

TCGA MapSplice aligned BAM files [59] were downloaded from TCGA for tumor and adjacent normal pairs from the following tissue types: breast invasive carcinoma (*n* = 104 pairs), colon adenocarcinoma (*n* = 16), head and neck squamous cell carcinoma (*n* = 37), kidney renal clear cell carcinoma (*n* = 69), stomach adenocarcinoma (*n* = 30), thyroid carcinoma (*n* = 58), and uterine corpus endometrial carcinoma (*n* = 10). A table comprising read counts for each transcript from our custom lncRNA annotation file was calculated using BEDTools version 2.17.0 [51]. Similar to the edgeR pipeline previously described, transcripts less than 200 nucleotides, with only one exon, or overlapping known pseudogenes were removed along with protein coding genes. Log fold changes were obtained from edgeR and FPKM [19] expression values were manually calculated as 10^9^(*M*/(*T* × *L*)) where *M* is the number of reads mapping to a transcript, *T* is the total number of mapped reads, and *L* is the transcript length. LncRNAs with FDR ≤10^-5^, absolute fold change ≥2, altered in the same direction as lung, and either average tumor FPKM or average normal FPKM ≥1 were called significantly differentially expressed.

### Association of *LCALs* with mutation status

The most frequently mutated genes in lung cancer were determined as having over 10% mutation rate in either the LUAD or LUSC cohort, as reported in Figure 2 from Kandoth *et al.* [44]. Mutation calls were downloaded from TCGA [60]. Attention was restricted to 167 LUAD and 178 LUSC samples with both RNA-Seq and mutation data. A Wilcoxon rank sum test was used to test for significance between mutational status and expression of each LCAL (using manually calculated FPKM values). For each mutated gene, *P*-values for the LCALs were corrected for multiple comparisons using the Benjamini and Hockberg FDR correction [61], and a significance threshold of 0.01 was used.

### Cell culture and human lung cancer RNA

A549, HOP62, HOP92, NCI-H522, -H32, -H460, -H322M, and -H226 were a kind gift from Dr Van Tine at Washington University. Calu-1, SK-MES-1, SW900, and HCC95 were a kind gift from Dr Loren Michel at Washington University. HCC827 was a kind gift from Dr Leonard Maggi at Washington University. BEAS-2B cells were purchased from American Type Culture Collection (Manassas, VA, USA). All cells were grown in RPM1-1640 (Invitrogen, Carsbad, CA, USA) with 10% fetal bovine serum and 1% penicillin/streptomycin. RNA (2 μg) from lung cancer tissue and their matched controls was obtained from the Tissue Procurement Core at Washington University.

### RNA isolation and cDNA synthesis

Total RNA was isolated with the RNeasy Mini Kit (QIAGEN) with DNase 1 treatment according to the manufacturer’s instructions. cDNA was synthesized from total RNA using High Capacity cDNA Reverse Transcription Kit with random hexamers (Invitrogen). Human lung cancer tissue RNA was used to make cDNA with the Superscript III RT-PCR Kit (Invitrogen).

### Quantitative real-time PCR

At least two biological replicates were used for qPCR using PowerSyBr Green (Invitrogen). The comparative CT (ΔΔCT) method was used with values first normalized to the housekeeping gene *RPL32*, and then to BEAS-2B control. All primers were obtained from Integrated DNA Technologies (Coralville, IA, USA) and are listed in Table S9 in Additional file 1. Primer efficiency between 90 and 110% was determined for each primer candidate.

### RACE

5’ and 3’ RACE was done using the GeneRacer Kit (Invitrogen) according to the manufacturer’s instructions. RACE PCR products were obtained with Platinum Taq High Fidelity (Invitrogen) using the GeneRacer primer (supplied) and a gene-specific primer (GSP) listed in Table S10 in Additional file 1. Nested PCR was also performed for most transcripts. Products were visualized on a 2% agarose gel and purified by gel extraction (QIAGEN). This product was then cloned into pcr4-TOPO vector (Invitrogen) and grown in TOP10 *Escherichia coli*. Clones were sequenced with the M13 forward primer at The Protein and Nucleic Acid Chemistry Laboratory at Washington University. Full-length sequences were uploaded to GenBank under the following accession numbers: KF773845 (*LCAL1*), KF773846 (*LCAL5*), KF773847 (*LCAL7*), and KF773848 (*LCAL80*).

### siRNA knockdown experiments

Stealth siRNA oligonucleotides were synthesized by Invitrogen. The following siRNA sequences were used for knockdown of *LCAL1*: siRNA 1 GGACAGGCTGCAGTCATCATATGGA and siRNA 2 GGCATGTGTTCAGACATATCCTAAA. Cells were transfected with 50 pmol of siRNA and a scrambled-matched %GC oligo as control with RNAimax Lipofecatmine (Invitrogen) following the manufacturer’s instructions. Knockdown efficiency was determined by qPCR at time of plating for assay. After 72 hours, cells were then plated at 200,000 cells/well for proliferation assays. Cells were counted using the Beckman Z1 Coulter Counter at days 2, 4, and 6. At least three biological replicates were performed for each siRNA construct over two experiments.

### Alamar Blue proliferation assays

Seventy-two hours after transfection, cells were seeded at 3,000 cells/well in a 96-well dish to assess viability via Alamar Blue according to the manufacturer’s instructions (Sigma). Subsequent cells were then used for RNA isolation to detect relative expression of *LCAL1*. Fluorescence intensity was then measured with Gen5 software on Synergy Hybrid (BioTek) at days 2, 4, and 6 post-knockdown after one hour incubation with Alamar Blue. At least four biological replicates were done for each siRNA construct over two experiments.

### Retroviral infection and generation of BEAS-2B cell lines stably expressing *LCAL1* variants

The full length *LCAL1* transcript was PCR amplified from H322M cells and cloned into the pCFG5-IEGZ vector (a kind gift from Dr Ron Bose). Full-length inserts were confirmed with Sanger sequencing at The Protein and Nucleic Acid Chemistry Laboratory at Washington University. Retroviral infection of BEAS-2B cells were performed according to Kavuri *et al*. [62]. Briefly, the amphotrophic producer cell lines were transfected with 10 μg of *LCAL1* and empty control retroviral vectors by calcium phosphate precipitation and incubated for 24 hours. Viral supernatants were harvested after an additional 24 hour incubation. Virus was added to BEAS-2B cells seeded in six-well dishes in the presence of 8 μg/ml Polybrene. BEAS-2B cells were centrifuged at 2,500 RPM for 1.5 hours at 22°C and supernatant exchanged for fresh media. After 10 to 14 days of 125 μg/ml zeocin selection, cells were plated at 200,000 cells/well for proliferation assays. Cells were counted using the Beckman Z1 Coulter Counter at days 2, 4, and 6. At least three biological replicates were performed for each stable cell line over two experiments. Cells were also collected for validation of *LCAL1* expression by qPCR.

### Nuclear localization

H322M lysates were fractionated into nuclear and cytosolic fractions according to the PARIS kit protocol (Invitrogen) and gene expression was assessed by qPCR. Results were normalized to the housekeeping gene *RPL32*, and then to total RNA. *U6* was used as a positive control for nuclear gene expression and *GAPDH* and *MT-RNR1* were used as positive cytoplasmic gene expression. Three biological replicates were conducted over two independent experiments.

## Additional files

Additional file 1:
**Supplementary Tables S1 to S10.**
Additional file 2:
**Supplementary Figures S1 to S10.**

## Abbreviations

ChIP-Seq: chromatin immunoprecipitation sequencing
FDR: false discovery rate
FPKM: fragments per kilobase of transcript per million mapped reads
GTF: gene transfer format
LCAL: lung cancer-associated lncRNA
lncRNA: long non-coding RNA
LUAD: lung adenocarcinoma
LUSC: lung squamous cell carcinoma
ORF: open reading frame
qPCR: quantitative real-time PCR
RACE: rapid amplification of cDNA ends
siRNA: short interfering RNA
TCGA: The Cancer Genome Atlas
