# Supplementary material for: Transcriptome sequencing reveals altered long intergenic non-coding RNAs in lung cancer
Source: Genome Biol. 2014 Aug 13;15(8):429. doi: 10.1186/s13059-014-0429-8 (PMC4156652; doi:10.1186/s13059-014-0429-8)

Supplementary Figure S1 | Characterization of unannotated transcripts. (A) CPAT coding probability scores and **(B)** cummulative distribution of ORF length are plotted for each category of protein-coding genes, known RNA genes, known lncRNAs, and novel lncRNAs. **(C)** Expression level distribution of novel lncRNA genes, known RNA genes, recently discovered lncRNAs, and protein-coding genes. **(D)** Repetitive content analysis of lncRNAs. Stacked bars represent the percentage of nucleotides covered by various transposable element families for known lncRNAs and novel lncRNAs.

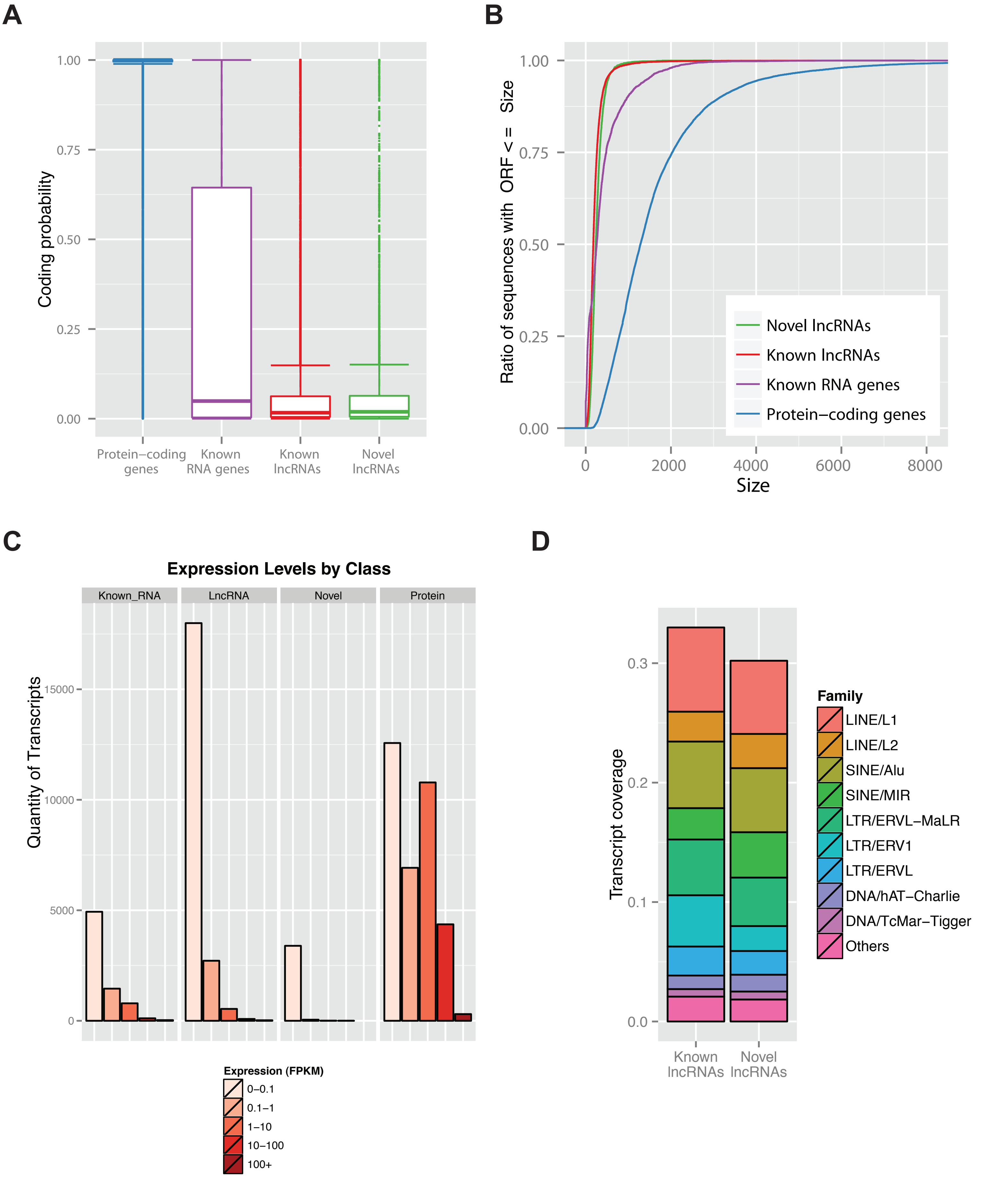

Supplementary Figure S2 | Differentially expressed lncRNAs in lung cancer. (A) Schematic showing the filtering steps used in our lncRNA differential expression pipeline. Heatmaps showing the differentially expressed lncRNAs in the **(B)** Seo, **(C)** LUAD, and **(D)** LUSC cohorts. Although only the paired tumors and normal tissues were used in the differential expression analysis, the expression of the unpaired tumors is also shown for the TCGA cohorts.

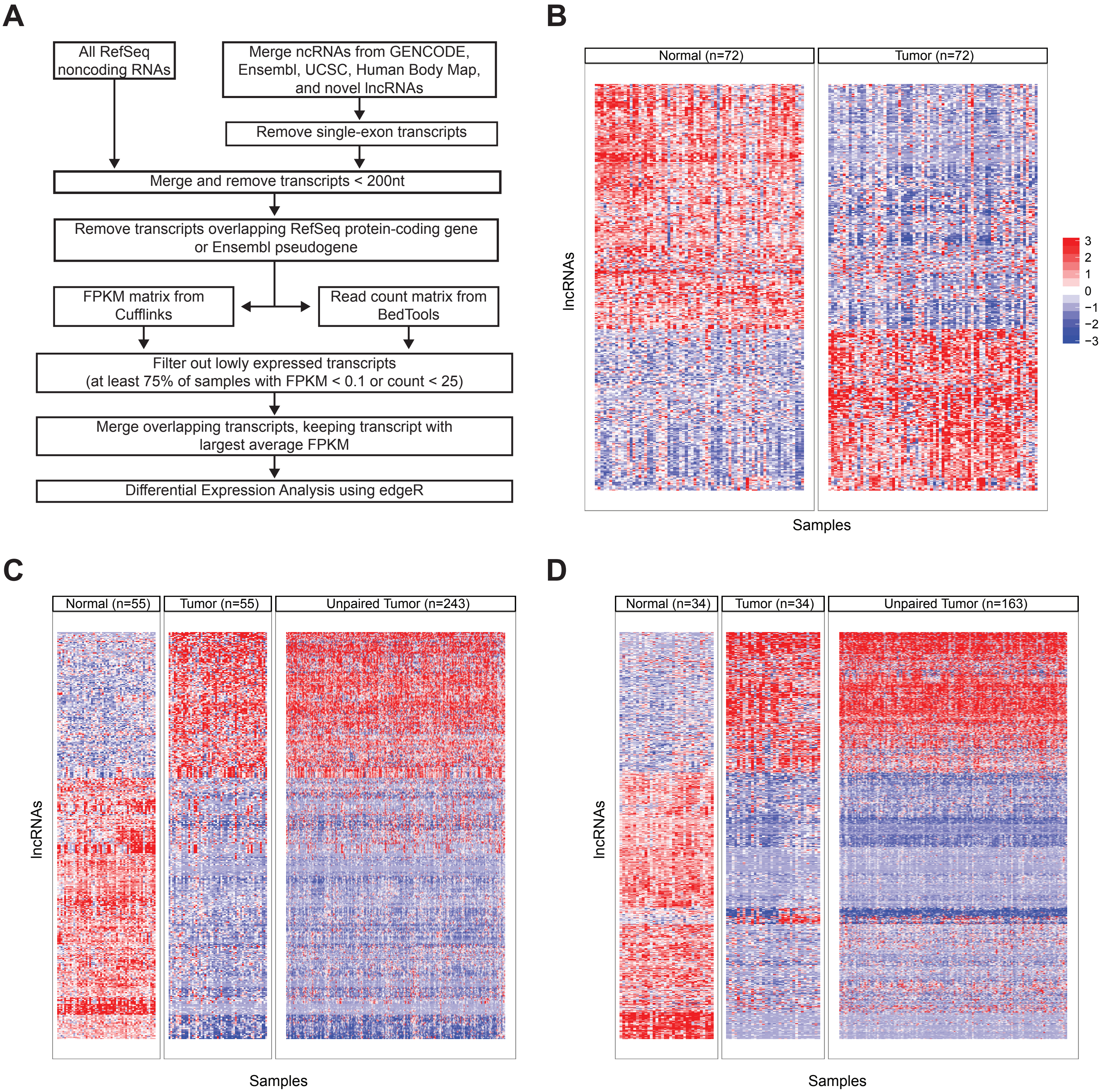

Supplementary Figure S3 | Subtype-specific lncRNAs. Heatmap of differentially expressed lncRNAs between TCGA lung adenocarcinoma (LUAD) and lung squamous cell carcinoma (LUSC) tumors.

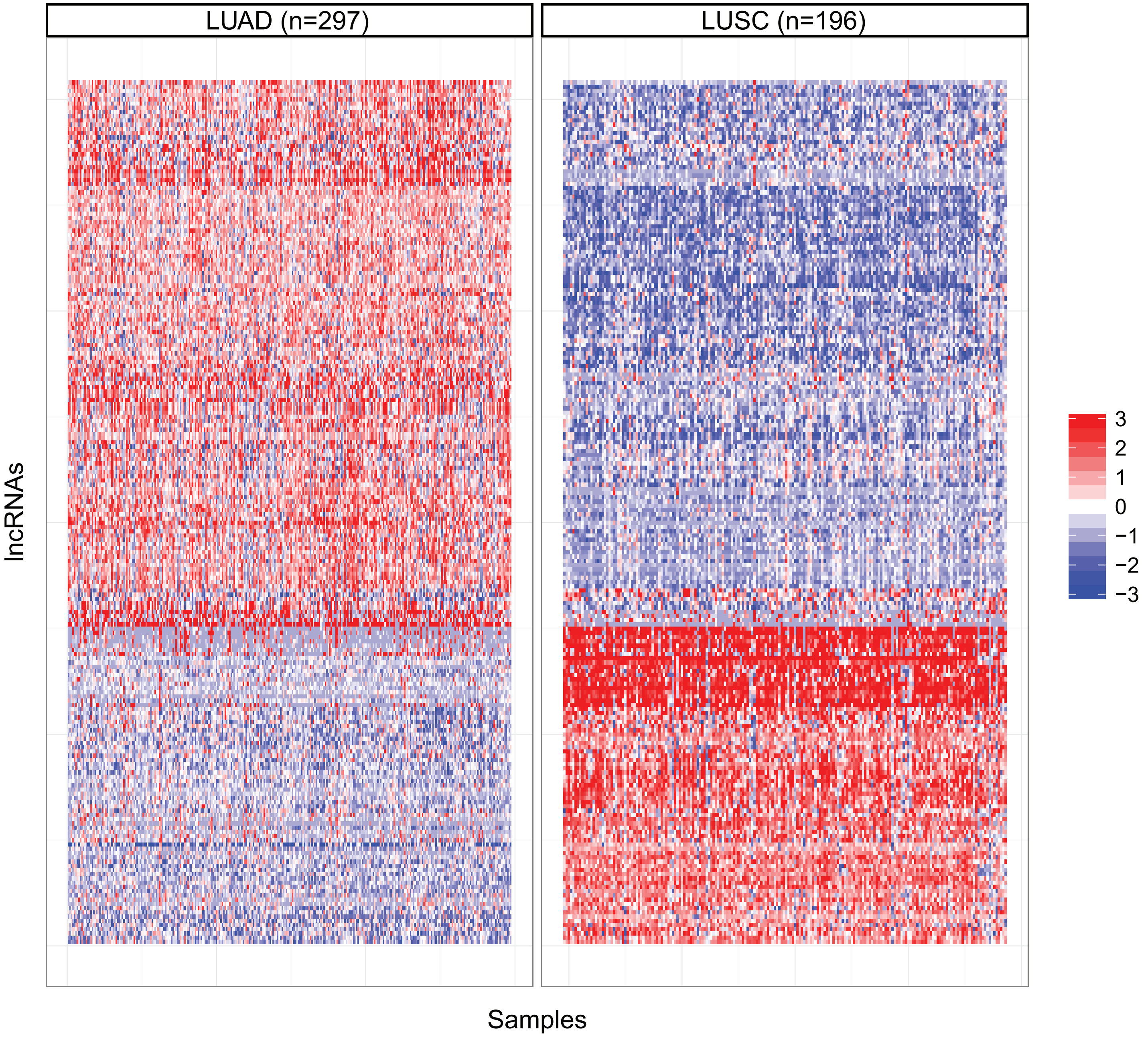

Supplementary Figure S4 | Lung cancer cell line validation. qPCR validation of six LCALs across a panel of lung cancer cell lines (n = 8) relative to a control cell line, BEAS-2B, and normalized to the housekeeping gene *RPL32*. All error bars are mean +/- standard error across n = 3 biological replicates.

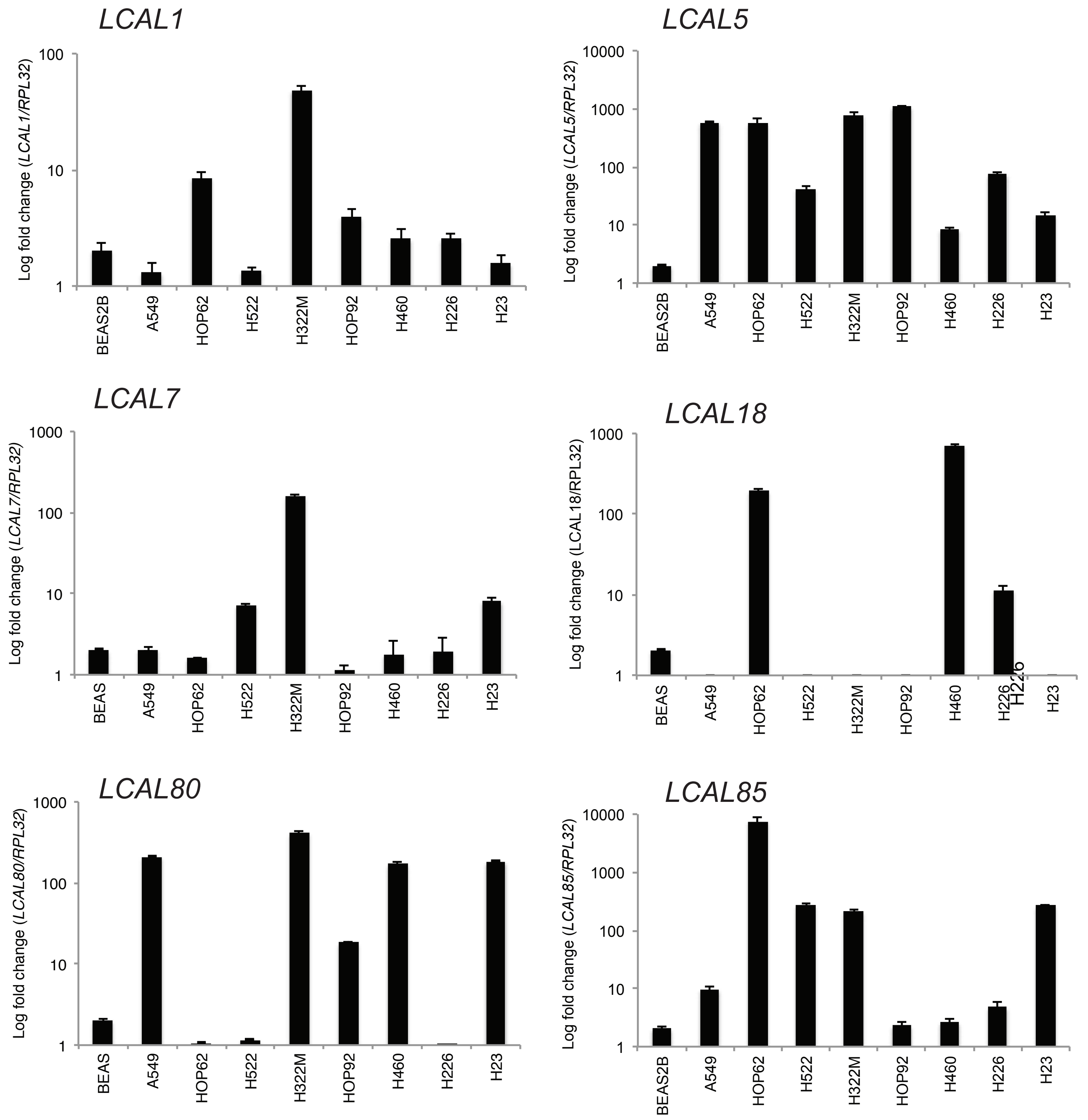

Supplementary Figure S5 | LCAL expression in lung cancer. Coverage maps showing the average expression levels of tumor and normal samples across all three lung cancer cohorts for **(A)** *LCAL18* (*FENDRR*) **(B)** *LCAL80* (*ESCCAL-1*; known as *CASC9* in RefSeq), and **(C)** *LCAL85* (*CCAT1*). Annotated RefSeq (dark blue), UCSC (light blue), and full-length transcripts as determined by 5′ and 3′ RACE in H322M cell line (black) are shown below each plot. qPCR validation in an independent cohort of human adenocarcinoma and matched controls and squamous cell carcinoma and matched controls are shown for **(D)** *LCAL18* **(E)** *LCAL80*, and **(F)** *LCAL85*. Insert tables distinguish "high" and "low" expression of LCALs using the cutoff value denoted by the dotted line. Because *LCAL80* and *LCAL85* are differentially expressed in the LUSC cohort only, the insert tables were calculated separately for the two subtypes. These results further demonstrate that *LCAL80* and *LCAL85* are broadly over-expressed in lung squamous cell carcinomas but not adenocarcinomas.

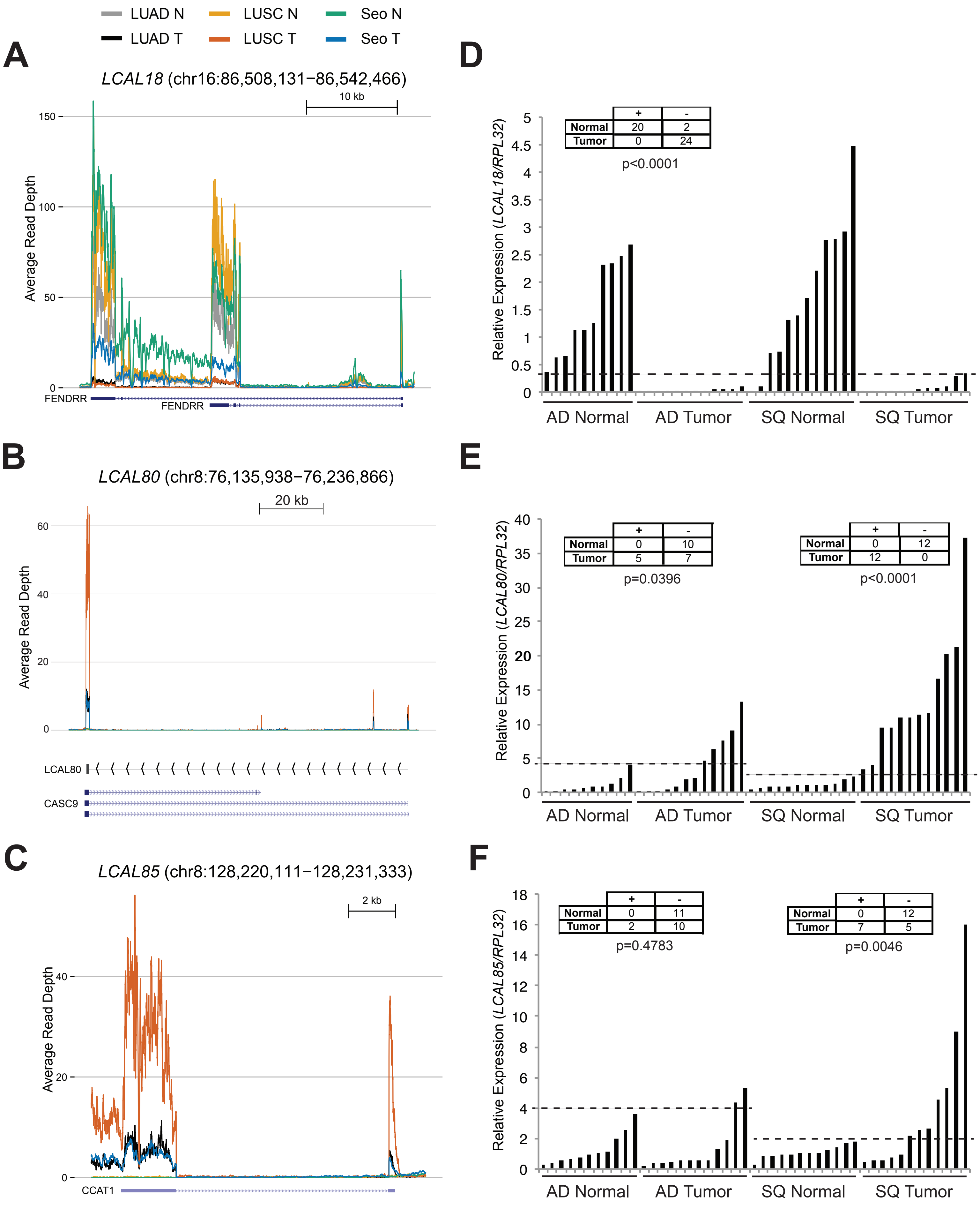

Supplementary Figure S6 | Association between LCAL expression and mutation status. Expression levels of **(A)** *LCAL1*, **(B)** *LCAL8*, **(C)** *LCAL24*, **(D)** *LCAL38*, **(E)** *LCAL74*, and **(F)** *LCAL84*, measured by log2 FPKM, for wild type (black) and mutant (colored) samples. Data points are ordered by expression levels and symbols designate cohort (squares for LUAD, circles for LUSC). Thick colored lines represent the median expression level for each group. P-values for each mutational association are also reported (*: FDR < 0.05, **: FDR < 0.01).

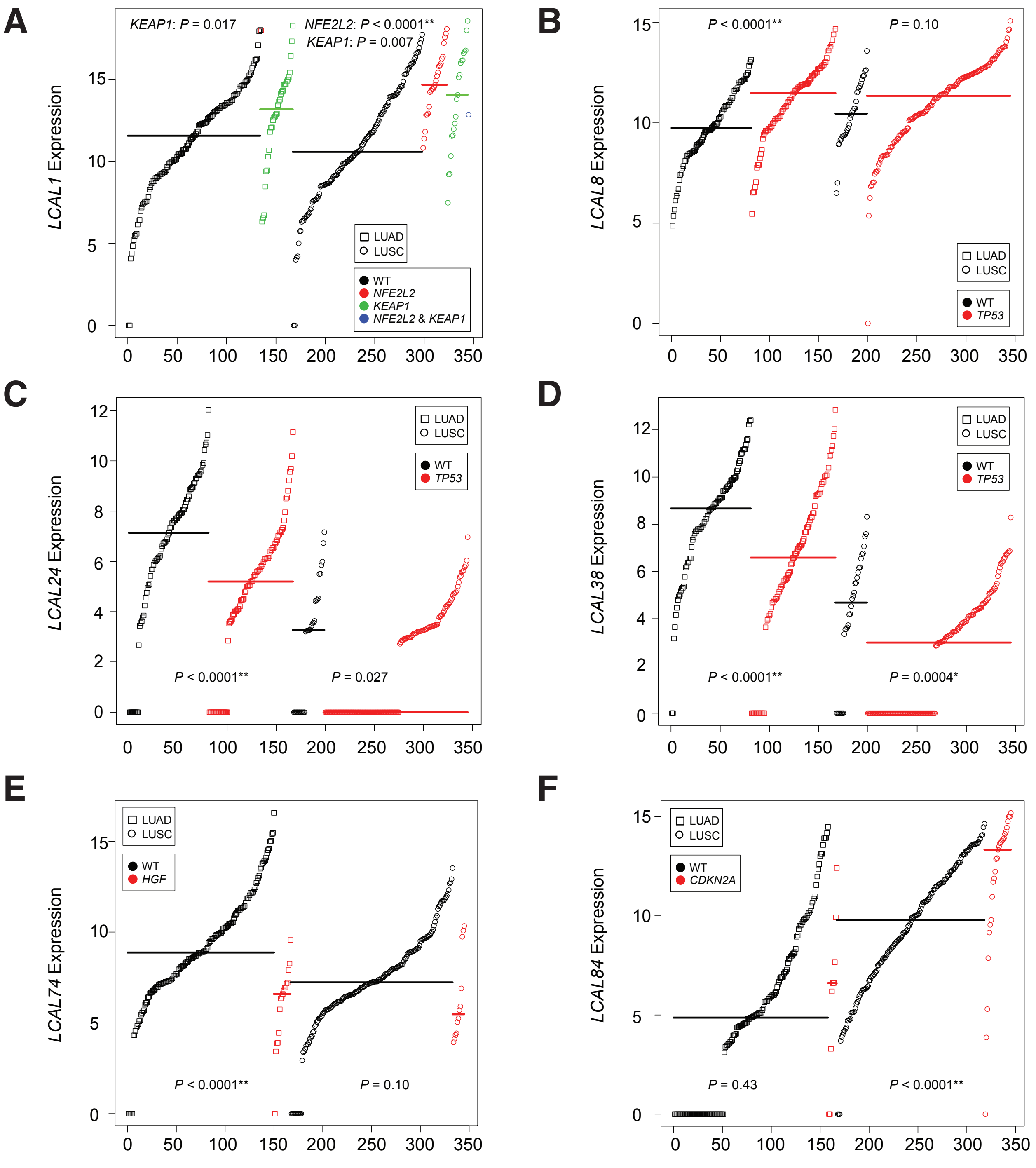

Supplementary Figure S7 | Conservation of *LCAL1*. The UCSC schematic shows a lack of Pfam domains or conserved RNA secondary structures predicted by EvoFold. ENCODE data shows DNaseI Hypersensitivity and transcription factor binding in the promoter of *LCAL1*. Evolutionary conservation, using PhyloP, does not show any strong basepair conservation within *LCAL1*. Multiz alignments across 100 vertebrates reveals *LCAL1* sequence similarity restricted within the majority of primates.

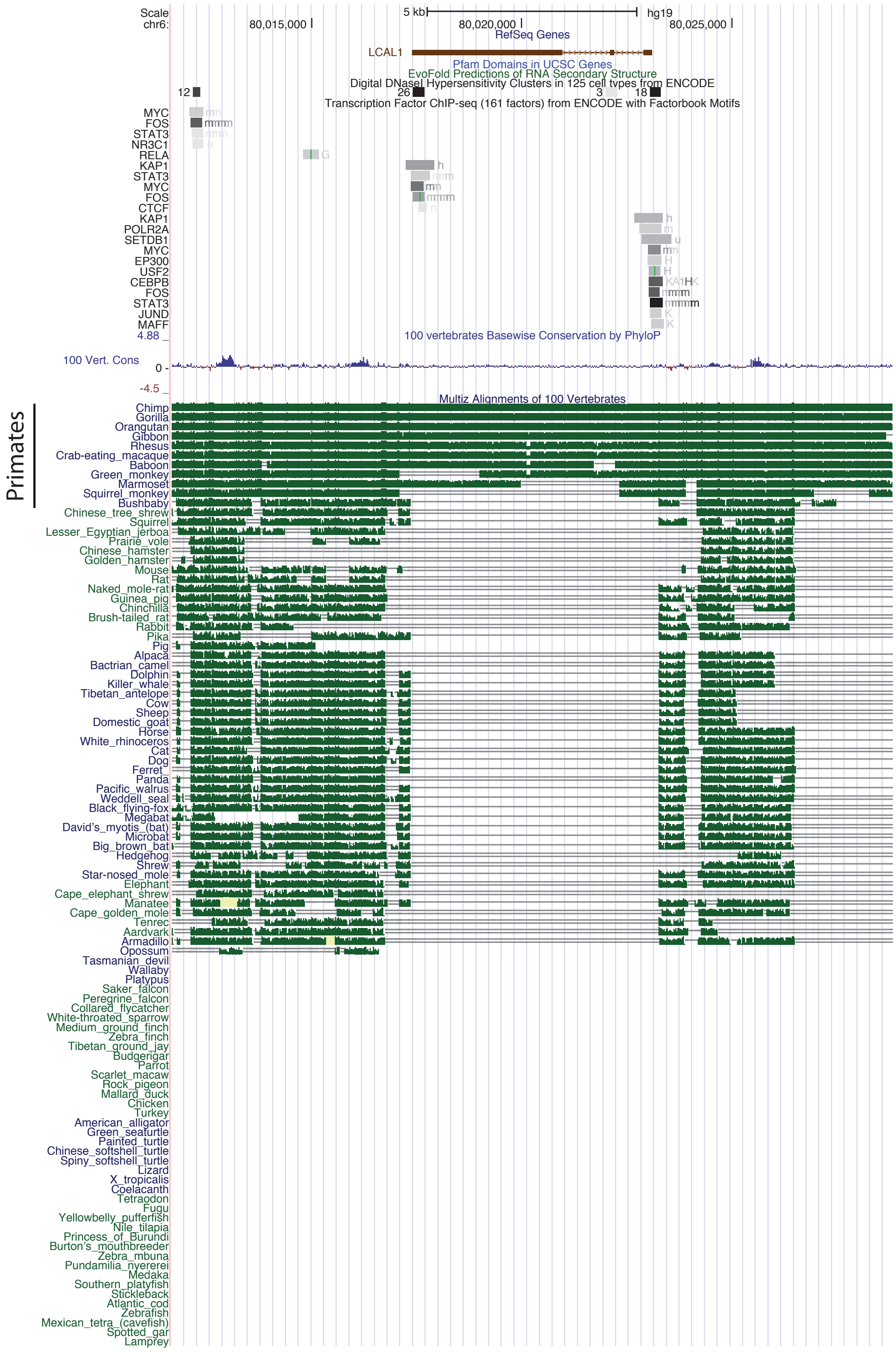

Supplementary Figure S8 | Nuclear Localization of *LCAL1*. Nuclear and cytosolic fractionation of lysates indicates high expression of *LCAL1* in the nucleus in H322M cells. *GAPDH* and *MT-RNR1* were used as positive control for cytosolic gene expression and *U6* was used as a positive control for nuclear gene expression. qPCR results are relative to total RNA and normalized to the housekeeping gene *RPL32*. All error bars are mean +/- standard error across three biological replicates in two independent experiments.

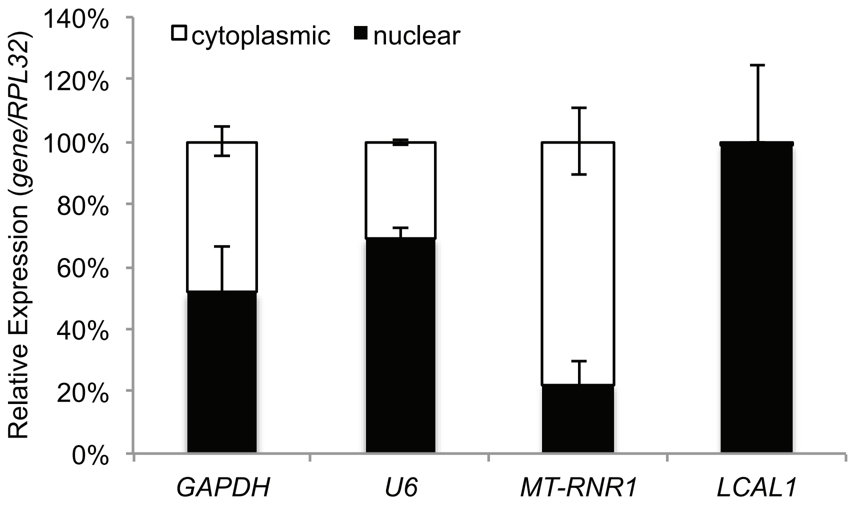

Supplementary Figure S9 | Lung cancer cell line validation. qPCR validation of *LCAL1* across a panel of squamous carcinoma cell lines (n=5) relative to a control cell line, BEAS-2B, and normalized to the housekeeping gene *RPL32*.

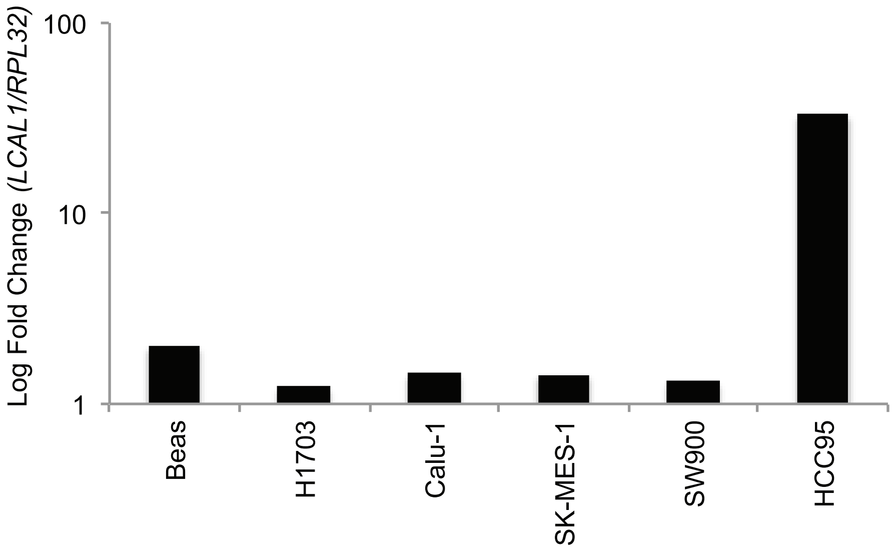

Supplementary Figure S10 | *LCAL1* expression affects cellular proliferation. After 72h transfection, cells were seeded in a 96-well plate at 3,000 cells/well. At indicated days Alamar Blue reduction was measured by fluorescence. Fluorescence was normalized to mean scrambled control. All error bars are mean +/- standard error across n=4 biological replicates in two independent experiments. * $P < 0.01$, ** $P < 0.001$, # $P < 0.0001$ by a two-tailed Student's t-test.

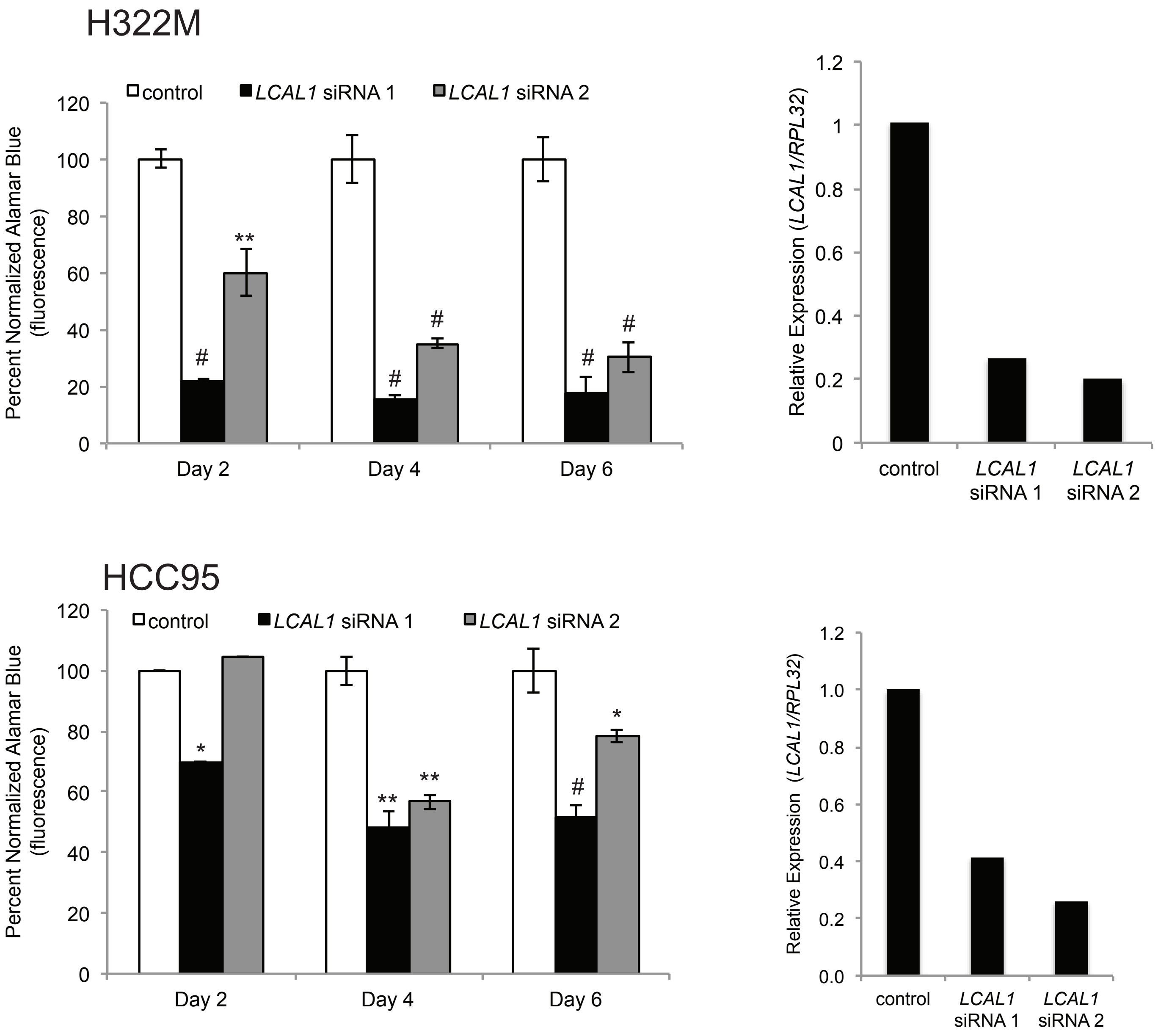

Supplement: Additional file 2: — Supplementary Figures S1 to S10. [file 13059_2014_429_MOESM2_ESM.pdf]
